# IMPACT OF COGNITION AND DEPRESSION ON PHYSICAL ACTIVITY IN OLDER ADULTS WITH CVD: NHANES 2011-2014

**DOI:** 10.1093/geroni/igae098.4216

**Published:** 2024-12-31

**Authors:** Hyun Jung Kim, Shweta Gore

**Affiliations:** MGH Institute of Health Professions, CAMBRIDGE, Massachusetts, United States; MGH Institute of Health Professions, Boston, Massachusetts, United States

## Abstract

Cardiovascular disease (CVD) is a major cause of morbidity and mortality. Physical activity (PA) is crucial for its prevention and management. Cognitive impairment and depression, common in older adults with CVD, impact PA and sedentary behaviors (SB). This study explores the associations between cognitive function, depression, PA, and SB in 632 older adults with CVD, using data from the NHANES 2011-2014. PA was assessed using the Global Physical Activity Questionnaire – meeting PA guideline as ≥ 500 MET-min/wk and SB as spending ≥ 7 hr/day in sedentary time. Cognitive function was measured as a composite z-score from four standardized tests (CERAD Word List Learning and Recall, Animal Fluency Test, and Digit Symbol Substitution Test). Depression was measured using the Patient Health Questionnaire (PHQ-9) and categorized into none (score: 0), minimal (1-9), moderate (10-14), and severe depression (15-27). Survey design methods were used to adjust for the complex sample design. Significant predictors from preliminary bivariate analyses were included in logistic regression models. When adjusting for covariates, higher cognitive function increased the odds of meeting PA guidelines by 79% (OR = 1.79, 95% CI = 1.01-3.17, p =.047) and moderate depression by 79% (OR = 0.21, 95% CI = 0.08-0.56, p =.003). Self-perceived general health was also a significant predictor of PA. On the contrary, cognitive function and depression were not significantly associated with SB. These results highlight the importance of considering cognitive and mental health to promote adherence of PA in this population.

